# Chitosan Combined with Poly-L-arginine as Efficient, Safe, and Serum-Insensitive Vehicle with RNase Protection Ability for siRNA Delivery

**DOI:** 10.1155/2013/574136

**Published:** 2013-06-23

**Authors:** Samarwadee Plianwong, Praneet Opanasopit, Tanasait Ngawhirunpat, Theerasak Rojanarata

**Affiliations:** Pharmaceutical Development of Green Innovations Group (PDGIG), Faculty of Pharmacy, Silpakorn University, Nakhon Pathom 73000, Thailand

## Abstract

Chitosan (CS) combined with poly-L-arginine (PLA) was formulated and evaluated for its performance to deliver siRNA to HeLa cells expressing enhanced green fluorescent protein (EGFP). Compared with the formulations using single polymer in which the polyplexes were completely formed at the weight ratio of >20 : 1 for CS/siRNA or 1 : 1 for PLA/siRNA, the combination of CS and PLA could reduce the amounts of the polymers required for the complete complexation with siRNA, thereby forming positively charged, nanosized polyplex at the weight ratio of CS/PLA/siRNA of 5 : 0.5: 1. In addition, while the transfection efficiency of CS/siRNA and PLA/siRNA was very low at physiological pH (7.4), CS/PLA/siRNA at the optimal weight ratio of 5 : 0.5 : 1 satisfactorily silenced the endogenous EGFP gene at pH 7.4 as well as at pH 6.4 without the deterrent effect from serum. The combined polymers could protect siRNA from RNase degradation over a period of at least 6 h. Furthermore, MTT assay results demonstrated that CS/PLA/siRNA complexes showed acceptably low cytotoxicity with 75% cell viability. Therefore, CS combined with PLA is easy to prepare, safe, and promising for use as an efficient siRNA delivery vehicle.

## 1. Introduction

The use of 21–23 mer duplex small interfering RNA (siRNA) molecules toinhibit the expression of specific genes is currently recognized as the potential means for the treatment of diseases such as cancers and genetic disorders [1–3]. However, the delivery of naked siRNA to the desired targets is usually limited by rapid degradation by nucleases and poor cellular uptake, leading to the low transfection efficiency [4–6] and the requirement to develop efficient vehicles for gene delivery formulations. Among the carriers investigated, cationic polymers effectively condense nucleic acids including siRNA via electrostatic association and assembly into polymer/siRNA complex called polyplex. Additionally, they promote the polyplex toescapefrom endosomal degradation throughproton sponge effect andcarrythe therapeutic materials intothe cytoplasm[7, 8]. Currently, cationic polymers such as polyethylenimine (PEI) and poly(amidoamine) (PAMAM) have been reported for siRNA delivery [9–12]. Although these polymers bind efficiently to nucleic acid, their highly positive charge may interact with negatively charged serum proteins and tissue components *in vivo*, therefore hindering the effective transfection. Moreover, the excessive positive charge may be toxic to cells.

Chitosan (CS) is a biodegradable and biocompatible natural polymer consisting of repeating units of D-glucosamine and N-acetyl-D-glucosamine. It is a weak base and is soluble only in acidic solution where the pH is lower than pKa (~6.5). Once the amino groups are protonated, CS binds and condenses negatively charged DNA or siRNA into smaller particles, enabling this polymer for use as gene delivery vehicle [13]. To date chitosan and its derivatives have been used to formulate with siRNA [14–19] where it has been shown that the optimal polyplex formation greatly depended on the weight ratio of CS/siRNA. Nevertheless, low complex stability and transfection efficiency are usually obtained at physiological pH when native chitosan was employed [13, 20–22].

Besides natural polymers, synthetic cationic polypeptides such as poly-L-lysine (PLL) [23–25] as well as poly-L-arginine (PLA) have also been used as gene carriers. Since PLA alone is known to be cytotoxic due to the highly positive charge and the ability to damage the mitochondria leading to the disturbance of cell metabolism and cell death [26], attempts have been made to resolve this drawback, for example, by mixing PLA with hyaluronic acid [27] or chemically conjugating it with CS [28] prior to use for siRNA delivery. The physical mixture of PLA and CS was previously reported to have the higher transfection efficiency and lower cytotoxicity than the polypeptide itself [29]. However, in that study such combined polymers were investigated for use to deliver plasmid DNA. Although siRNA and plasmid DNA share some common properties that they are both double-stranded nucleic acids with anionic backbones to interact with cationic agents, upon a deeper look these two molecules possess distinct characteristics. In term of molecular topography, although siRNA is smaller molecule, its rigid rod shape may result in incomplete encapsulation and/or undesirably large polyplex size when mixed with improper proportions of polymer. Moreover, unlike DNA which contains the sugar deoxyribose, the RNA backbone contains ribose which has a hydroxyl group in the 2′ position of the pentose ring instead of a hydrogen atom and this extra hydroxyl group makes the siRNA more susceptible to hydrolysis by serum nucleases [7]. By these reasons, the delivery strategies should be developed to suit each case individually. However, until now there have not been any reports about the use of easy-to-prepare CS combined with PLA formulations for siRNA delivery. In this study, the mixtures consisting of different ratios of these two polymers were therefore formulated and evaluated for their performance to form the polyplex with siRNA. In addition to the characterization of the size and surface charge, the resulting polyplexes were tested for *in vitro* transfection efficiency with HeLa cells expressing stable and constitutive enhanced green fluorescent protein (EGFP) at the different pH and in the presence of serum. Furthermore, their RNase protection ability and cytotoxicity were investigated.

## 2. Experimental

### 2.1. Materials

Chitosan was purchased from Seafresh Chitosan Lab., Thailand, with molecular weight (MW) of 45 kDa and 85% degree of deacetylation. Polyethylenimine (PEI; MW 25 kDa) was purchased from Aldrich (Milwaukee, WI). PLA hydrochloride (MW of >70,000 Da), agarose, diethylpyrocarbonate (DEPC), 3-(4,5-dimethylthiazol-2-yl)-2,5-diphenyl tetrazolium bromide (MTT), and RNase A were purchased from Sigma (St. Louis, MO, USA). Modified Eagle's medium (MEM), fetal bovine serum (FBS), Trypsin-EDTA, and penicillin-streptomycin were purchased from Gibco BRL (Rockville, MD, USA). siRNA-EGFP(+) and siRNA-EGFP(−) were synthesized by using Ambion's Silencer siRNA Construction Kit (Ambion, USA). HeLa cells, a human cervical carcinoma cell line, were obtained from American Type Culture Collection (ATCC, Rockville, MD, USA). All other chemicals were of cell culture and molecular biology grade.

### 2.2. Preparation of siRNA

The EGFP targeted siRNA, siRNA-EGFP(+), and the mismatch siRNA, siRNA-EGFP(−), were synthesized using Ambion's Silencer siRNA Construction Kit (Ambion, USA). The duplex siRNA-EGFP(+) contains sense 5′-gcu gac ccu gaa guu cau cuu-3′ and antisense 5′-gau gaa cuu cag ggu cag cuu-3′. The siRNA-EGFP(−) contains sense 5′-gca ccg cuu acg uga uac uuu-3′ and antisense 5′-agu auc acg uaa gcg gug cuu-3′. The siRNA-EGFP(+) targets to position 124–144 of EGFP open-reading frame. The mismatch siRNA-EGFP(−) were designed by scrambling the nucleotide sequence of siRNA-EGFP(+) and performing blast analysis to identify the sequences that lack of significant homology to human genes.

### 2.3. Polyplex Formation

CS was dissolved in 0.01 mM hydrochloric acid prepared in DEPC-treated water to a final concentration of 1 mg/mL (stock solution). PLA was dissolved in DEPC-treated water to a final concentration of 1 mg/mL (stock solution). Purified siRNA from the construction reaction was diluted to 2 **μ**M in DEPC-treated water. To prepare the CS/siRNA complex, the varied amounts of CS solution were added to siRNA solution of fixed concentration to obtain the different weight ratios ranging from 0.1 : 1 to 20 : 1. The PLA/siRNA complex was formed in a similar manner by adding PLA solution to siRNA solution at the different weight ratios of 0.01 : 1 to 5 : 1. To form the CS/PLA/siRNA complex, the PLA solution was added to siRNA solution to obtain the PLA/siRNA with the weight ratios of 0.01, 0.05, 0.1, 0.5, 1, and 5. After 5 min, CS solution was subsequently added to PLA/siRNA solution to obtain the fixed CS weight ratio of 5. In all cases, the final mixtures were gently mixed by using micropipettes and the complex was allowed to form at room temperature for 30 min.

### 2.4. Agarose Gel Electrophoresis

The polyplex formation was confirmed by gel retardation assay using agarose gel electrophoresis. Briefly, the mixtures of 10 **μ**L of the complex containing 140 ng of siRNA and 2 **μ**L of 50% glycerol in water were loaded in the wells on 1% agarose gel containing 0.5 **µ**g/mL ethidium bromide. The electrophoresis was carried out at 100 V in Tris borate-EDTA (TBE) running buffer of pH 8.3 for 20 min. The siRNA in the complexes were visualized under a UV transilluminator using a GelDoc system.

### 2.5. Size and Zeta Potential Measurement

The size and zeta potential of CS/siRNA, PLA/siRNA, and CS/PLA/siRNA polyplexes were measured using the Zetasizer Nano ZS (Malvern Instruments Ltd., Malvern, UK) at room temperature (25°C). The samples of polyplexes diluted with sterile water were previously adjusted to pH 6.4 or 7.4 before the zeta potential measurement. All samples were measured in triplicate.

### 2.6. Preparation of HeLa Cells Stably Expressing Green Fluorescent Protein

HeLa cells with stable constitutive expression of EGFP were generated. Briefly, HeLa cells were transfected with pEGFP-C2 plasmids (Clontech, USA) using lipofect-amine2000 (Invitrogen, USA). The cells were trypsinized, diluted, and transferred to a 10 cm diameter plate to get a few hundred cells. Several single cells were marked under the plate and treated with 0.5 mg/mL G418 to generate clones of stable cell lines. Individual clones were isolated and expanded in modified Eagle's medium (MEM), supplemented with 10% fetal bovine serum (FBS), and treated with 0.1 mg/mL G418 every 3-4 weeks to maintain the EGFP gene expression.

### 2.7. *In Vitro* Gene Silencing Experiments

The HeLa cells expressing EGFP were cultivated in modified Eagle's medium (MEM), supplemented with 10% FBS, 2 mM L-glutamine, and 1% nonessential amino acid solution in a humidified atmosphere (5% CO_2_, 95% air, 37°C). The cells were trypsinized and seeded at the density of 9,000 cells/well in black clear-bottom, 96-well plates (Corning, USA) for 24 hours prior to transfection. Measurement of EGFP was performed using a fluorescence microplate reader (Universal Microplate Analyzer, Model AOPUS01 and AI53601, Packard Bio-Science, CT, USA) with excitation/emission at 485/530 nm. The seeding variation as measured by the fluorescence intensity of each well before transfection was less than 10%. The CS/siRNA-EGFP(+) or siRNA-EGFP(−), the PLA/siRNA-EGFP(+) or siRNA-EGFP(−), and the CS/PLA/siRNA-EGFP(+) or siRNA-EGFP(−) complexes with various weight ratios were formed as described in the complex formation. The PEI/siRNA-EGFP(+) or siRNA-EGFP(−) complexes at weight ratio of 2 were transfected as a control. On the day of transfection, the cells were washed with PBS and added with the mixtures of 75 **μ**L serum-free medium (MEM containing 100 U/mL penicillin G and 100 **µ**g/mL streptomycin) and 25 **μ**L of complexes, containing 210 ng of siRNA. In the case of CS/siRNA transfection, the transfection experiment was done in the transfection medium pH 6.4 and 7.4. After 6 hours of transfection, the transfection medium was removed and replaced by serum-rich medium containing antibiotics. Since the highest silencing efficiency was observed at the posttransfection period of 4 days for all polymers/siRNA formulations, plates were incubated for 4 days at 37°C under 5% CO_2_ condition. The culture medium was changed every other day and the fluorescence intensity was measured daily.

To take into account the variation of fluorescent intensity due to unequal initial number of cells in each well, % seeding variation was first calculated by (1) and then used to adjust the fluorescent intensity obtained from each well by (2). The percentage of EGFP gene silencing was calculated by (3). Consider

(1) $$\begin{array}{c} \%\;\text{seeding}\;\text{variation}=\frac{\left(I_{\text{average},\text{day}0}-I_{n,\text{day}0}\right)}{I_{\text{average},\text{day}0}}\times 100, \end{array}$$

(2) adjusted  fluorescent  intensity(Iad) =In,day1–4+[(In,day1–4)×(%  seeding  variation)100],

(3) $$\begin{array}{c} \%\;\text{EGFP}\;\text{gene}\;\text{silencing}=\frac{\left(I_{\text{ad},\text{siRNA}(-)}-I_{\text{ad},\text{siRNA}(+)}\right)}{I_{\text{ad},\text{siRNA}(-)}}\times 100, \end{array}$$

where *I*_average,day0_ is the average fluorescent intensity of all wells prior to transfection (day 0), *I*_*n*,day0_ is the fluorescent intensity of individual well at day 0, *I*_*n*,d1−4_ is the fluorescent intensity at days one through four of each well, *I*_ad_ is adjusted fluorescent intensity, *I*_ad,siRNA(+)_ is adjusted fluorescent intensity of individual well with polymers/siRNA(+) complexes, and *I*_ad,siRNA(−)_ is the average value of adjusted fluorescent intensity of 3 wells with polymers/siRNA(−) complexes.

To observe the interfering effect of serum on the transfection efficiency, the protocol was the same as described earlier, except that the transfection medium of MEM with 10% FBS was used instead of the serum-free MEM medium. All the gene silencing assays were carried out in triplicate.

### 2.8. Stability Study of siRNA in the Presence of RNase

The protection ability of CS/PLA/siRNA complex against RNase digestion was investigated. Briefly, the optimal CS/PLA/siRNA formulation (weight ratio of 5 : 0.5 : 1) containing of 0.21 **µ**g of siRNA was incubated with RNase (5 mIU/**µ**g of siRNA) in phosphate buffer (pH 7.0) at 37°C. After the various time periods (0, 0.5, 1, 2, 4, and 6 h), the samples were subjected to RNase inactivation at 70°C for 30 min. The siRNA was displaced from the polymers by incubating the sample with 3 **µ**L of 16 mM sodium dodecyl sulfate (SDS) solution at room temperature for 10 min. The extracted siRNA was analyzed on 1% agarose gel electrophoresis carried out at 100 V for 20 min. A positive control containing naked siRNA was tested under the same conditions.

### 2.9. Cell Viability Assay

To investigate the cytotoxicity of the formulations, cell viability upon the transfection of polymers/siRNA polyplexes was determined by MTT assay. EGFP-HeLa cells in 100 **µ**L culture medium were seeded at a cell density of 9,000 cells per well in 96-well plates and incubated for 24 h prior to transfection with polymers/siRNA polyplexes in serum-free medium at the same concentrations as *in vitro* transfection experiment. After 6 h of transfection, the medium was replaced with the serum-rich medium and continually incubated for 24 h. Subsequently, 20 **μ**L of MTT (5 mg/mL) was added to each well and the incubation was continued for 4 h. The medium was then removed, the cells were rinsed with PBS pH 7.4, and formazan crystals formed in the living cells were dissolved in 100 **μ**L DMSO per well. Relative viability (%) was calculated based on absorbance at 550 nm using a microplate reader (Universal Microplate Analyzer, Model AOPUS01 and AI53601, Packard BioScience, CT, USA). The viability of nontreated control cells was arbitrarily defined as 100%.

### 2.10. Statistical Analysis

Data are presented as the means ± standard deviations (SD) for three experiments. The statistical significance of gene silencing efficiency and cell viability were determined using one way analysis of variance (ANOVA) followed by an LSD post hoc test. *P* values of <0.05 were considered significant.

## 3. Results and Discussion

### 3.1. Formation of Polymers/siRNA Polyplexes and Their Characteristics

**Figure 1:**
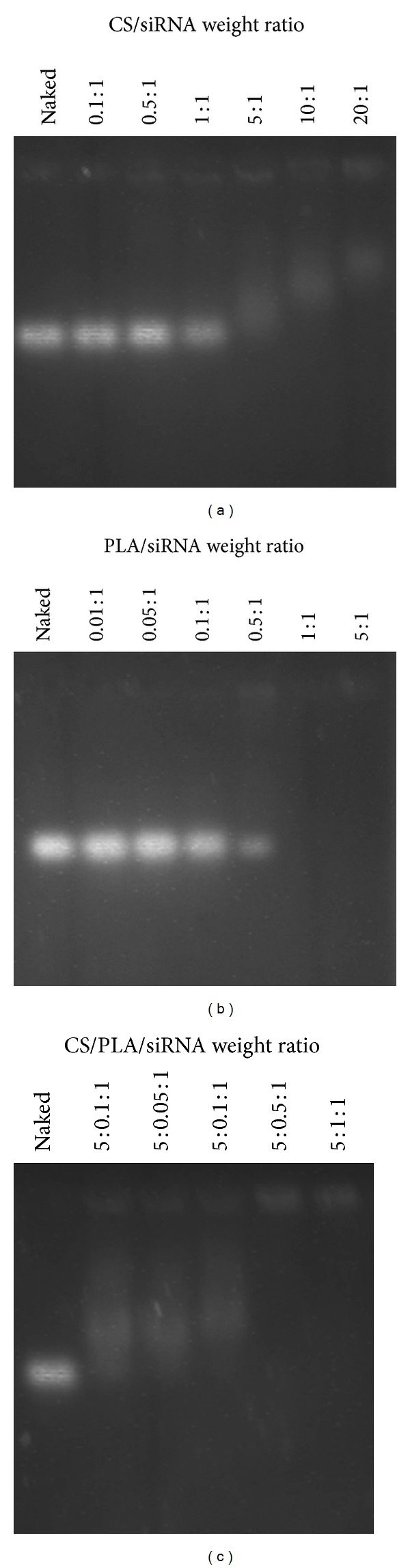
The agarose gel electrophoresis of polymers/siRNA complexes formulated by (a) CS, (b) PLA, and (c) CS/PLA at various weight ratios.

The association of the polymer with siRNA to form the polyplexes was observed by the retardation of siRNA bands on agarose gel electrophoresis. As showed in Figure 1(a), CS could not form the complex completely with siRNA even at the maximum weight ratio tested (20 : 1). PLA could bind to siRNA at the higher extent when the increasing amounts of polymer were used and it completely formed the polyplex, as seen by the absolute disappearance of band, at the weight ratio of 1 : 1 (Figure 1(b)). Interestingly, when CS/PLA/siRNA polyplex formation experiments was conducted by using PLA at different weight ratios ranging from 0.01 to 1 and CS at the fixed weight ratio of 5, the complete polyplex formation occurred at the lower CS/PLA/siRNA weight ratio of 5 : 0.5 : 1 (Figure 1(c)). This result indicated that the combination of PLA and CS could reduce the amount of each polymer required for the polyplex assembly.

**Figure 2:**
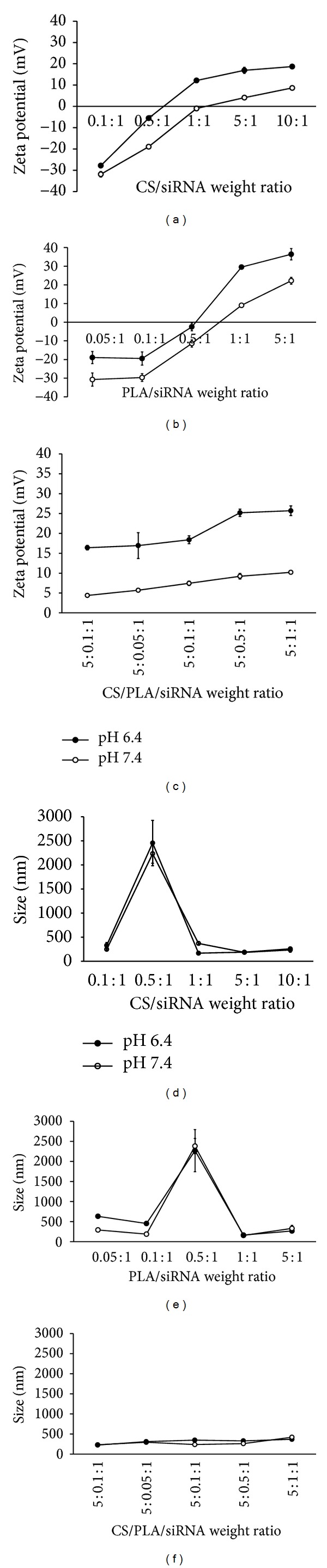
Zeta potential ((a)–(c)) and size ((d)–(f)) at pH 6.4 (filled circle) and pH 7.4 (blank circle) of the polyplexes at various weight ratios. Data are presented as the mean ± SD (*n* = 3).

Figures 2(a)–2(c) show the effect of polymer/siRNA ratio on the surface charge of the polyplexes. It was found that the zeta potential values increased with the increasing ratio of cationic polymers. For all polyplexes, the surface charge at pH 6.4 was higher than that at pH 7.4 because the polymers were protonated at a higher extent at this acidic pH. In case of the polyplexes formed by using the combined polymers, for example, CS/PLA/siRNA (5 : 0.5 : 1), the charge of the resulting polyplexes at pH 7.4 (~+10 mV) was higher than that of the polyplexes formed by using the same quantity of CS but in absence of PLA (CS/siRNA; 5 : 1; ~+4 mV). This should be the result from the positive charge of PLA which was incorporated into the polyplex, therefore maintaining the positive charge on the polyplexes surface at this pH.

In terms of size (Figures 2(d)–2(f)), CS/siRNA polyplexes were larger when the weight ratios increased from 0.1 : 1 to 1 : 0.5 and were then smaller at the weight ratios of ≥1 : 1. The zeta potentials of CS/siRNA complexes continuously increased and reached the positive values at the weight ratios of ≥1 : 1 at pH 6.4 and ≥1 : 5 at pH 7.4. The similar trend was also observed on the size of PLA/siRNA. In the case of CS/PLA/siRNA, the sizes of polyplexes were relatively constant with the range of 200–400 nm. 

### 3.2. *In Vitro* Transfection Efficiency

**Figure 3:**
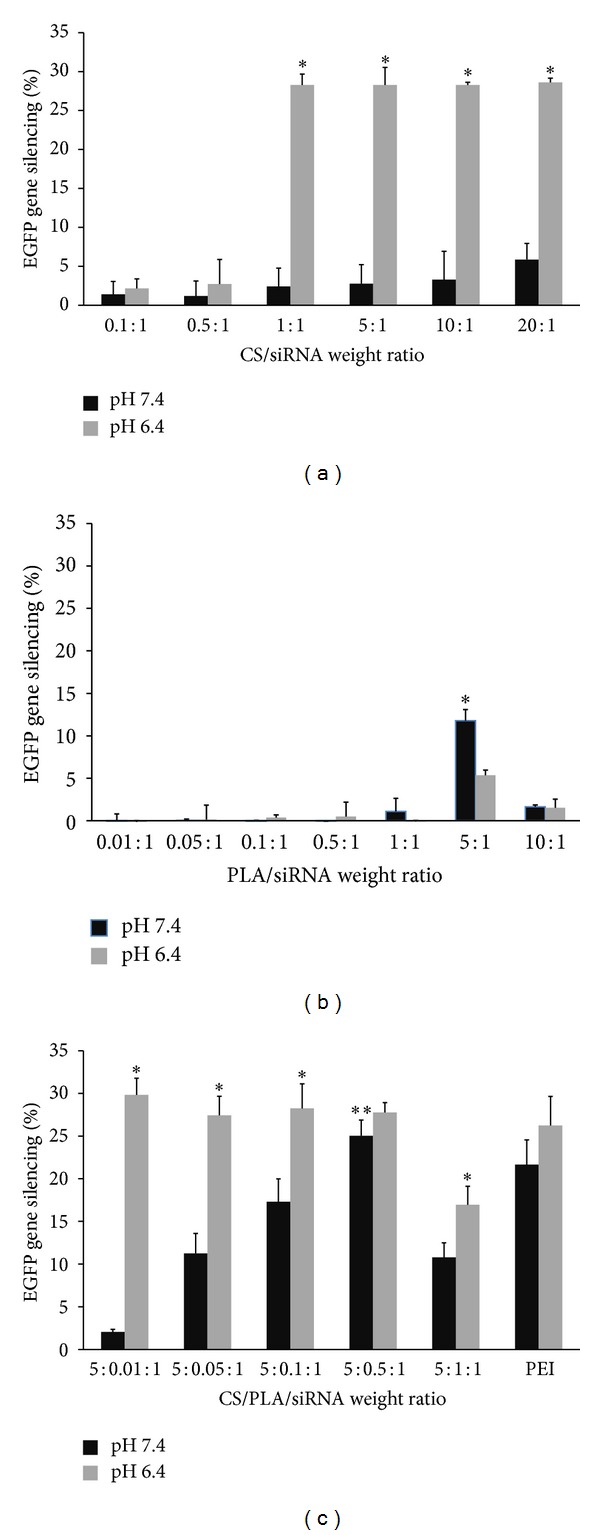
Percentage of EGFP gene silencing by (a) CS/siRNA, (b) PLA/siRNA, and (c) CS/PLA/siRNA polyplexes at various weight ratios in HeLa cells stably expressing EGFP after 4 days posttransfection at pH 7.4 (black bars) and pH 6.4 (gray bars). PEI (25 kDa)/siRNA polyplex at weight ratio of 2 : 1 was used as a positive control. Data are plotted as mean ± SD (*n* = 3). *Significantly different from the group(s) treated with the same weight ratio at pH 7.4. **Significantly different from the group(s) treated with the different weight ratio at same pH.

The transfection efficiency of the prepared polyplex was evaluated by the capability of silencing EGFP gene expressed in HeLa cells. The transfection with polymers/siRNA(−) complexes containing EGFP-mismatch siRNA was also performed in parallel with polymers/siRNA(+) complexes to confirm the knockdown specificity. The experimental results revealed that naked siRNA showed negligible gene silencing effect. The positive control (PEI/siRNA) at the optimal weight ratio of 2 : 1 showed the gene silencing efficiency of 21.66 ± 2.90% and 26.24 ± 3.40% at pH 7.4 and 6.4, respectively, in the absence of serum. Generally, the silencing activity of the polyplexes depended on the weight ratio of the polymers and siRNA and the pH of the transfection medium. At pH 6.4 where the amines of CS were protonated, CS/siRNA polyplex produced the highest gene silencing of about 28% at CS/siRNA ratio of ≥1 : 1 (Figure 3(a)). The polyplexes prepared at these ratios showed the positive charge on their surface (Figure 2(a)). At the physiological pH (7.4), however, the transfection efficiency of CS/siRNA polyplexes was drastically dropped, probably due to the lower degree of protonation which resulted in the lower positive charge. In case of PLA, it could not form the complete PLA/siRNA polyplex if used at the low ratio and without CS. From the experiments, the maximum gene silencing effect of PLA/siRNA occurred at the weight ratio of 5 : 1. Nevertheless, this silencing effect of 5 : 1 PLA/siRNA was lower than that of 5 : 1 CS/siRNA because of the higher cytotoxicity of PLA than CS. This is confirmed by the finding that the use of PLA/siRNA at the higher weight ratio than 5 : 1 (e.g., 10 : 1) did not further increase the gene silencing effect, but adversely lowered this silencing effect.

**Figure 4:**
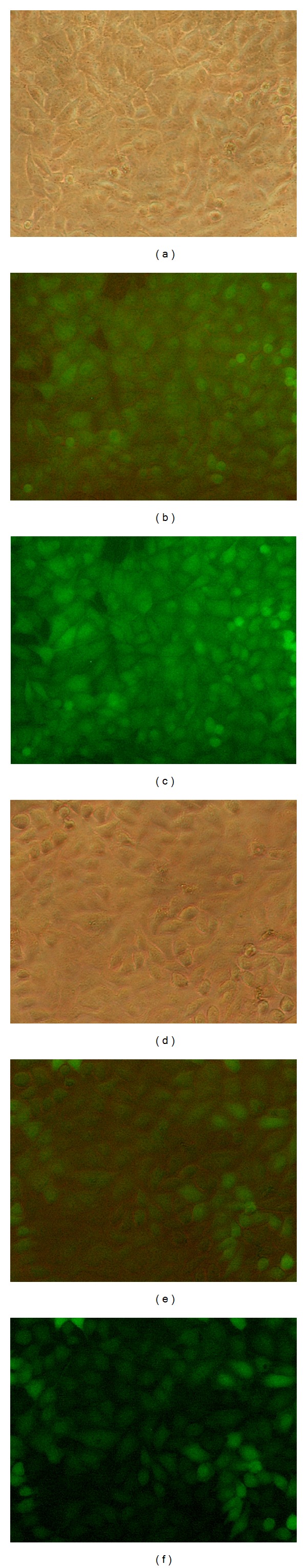
Microscopic images of EGFP expressing cells which were not transfected with polyplexes (control; (a)–(c)) versus cells transfected with CS/PLA/siRNA at the weight ratio of 5 : 0.5 : 1, pH 7.4 ((d)–(f)). The images were observed under bright field ((a) and (d)), merge field ((b) and (e)), and fluorescence field ((c) and (f)) microscopy at the magnification of 100x.

To overcome the problems associated with low and pH-dependent transfection efficiency, the combination of CS and PLA was tested for use as vehicle by mixing siRNA with varied weight ratios of PLA/siRNA from 0.01 : 1 to 1 : 1, followed by the addition of CS at fixed CS/siRNA ratio of 5 : 1. The 5 : 1 CS/siRNA was chosen because the polyplex formed at this ratio was smaller than that obtained from the weight ratio of 1 : 1 and gave the similar gene silencing effect. As shown in Figure 3(c), the expression EGFP gene was satisfactorily inhibited at pH 6.4 by CS/PLA/siRNA complexes prepared at the ratio of 5 : 0.01 : 1 to 5 : 0.5 : 1 and this effect was comparable to that of PEI. Moreover, the optimal amount of PLA which was not significantly cytotoxic could promote the positive charge of the polyplexes at pH 7.4 and recovered the gene silencing activity. The maximum reduction of EGFP gene expression of 25.04 ± 1.84% was achieved at this pH when 5 : 0.5 : 1 ratio of CS/PLA/siRNA was used.  Figure 4 illustrates the gene silencing effect as seen by the reduction of fluorescence upon the transfection with CS/PLA/siRNA polyplex (5 : 0.5 : 1) at pH 7.4 compared to the nontransfected cells. These results indicated that although PLA itself was not an effective gene carrier, however, when combined with CS, it probably helped provide the positive charge to the polyplexes at pH 7.4 where CS alone could be protonated at the low extent.

**Figure 5:**
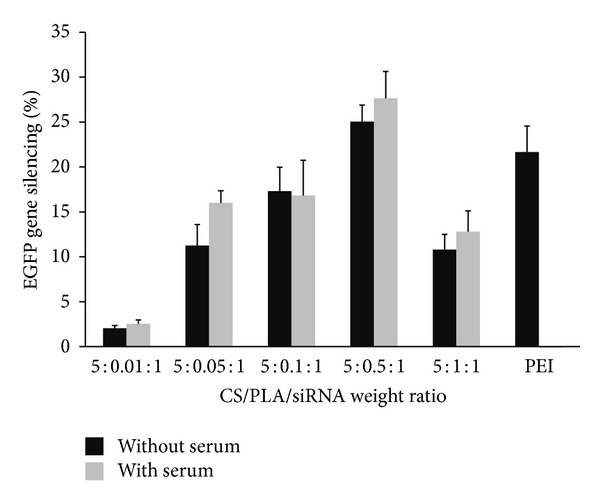
Comparison of percentage of EGFP gene silencing by CS/PLA/siRNA complexes at various weight ratios in HeLa cells stably expressing EGFP after 4 days posttransfection at pH 7.4 between in the absence of serum (black bars) and the presence of serum (gray bars). The PEI (25 kDa)/siRNA at weight ratio of 2 : 1 was used as a control. Data are plotted in mean ± SD (*n* = 3).

Since the susceptibility to serum is another problem which hampers the successful gene delivery by several cationic polymers, the transfection of polymers/siRNA complexes in the presence of serum (10% FBS) was also investigated in this study. As shown in Figure 5, the polyplex made from CS combined with PLA could silence the expression of EGFP gene in the medium containing serum equivalently to that without serum. In contrast to the polyplex formed by using PEI which has been known to be serum-sensitive polymer, the gene silencing was totally abolished when the transfection was conducted in the presence of serum. These results encouraged the further use of CS/PLA vehicle for siRNA delivery *in vivo*.

### 3.3. RNase Protection Ability of CS/PLA

**Figure 6:**
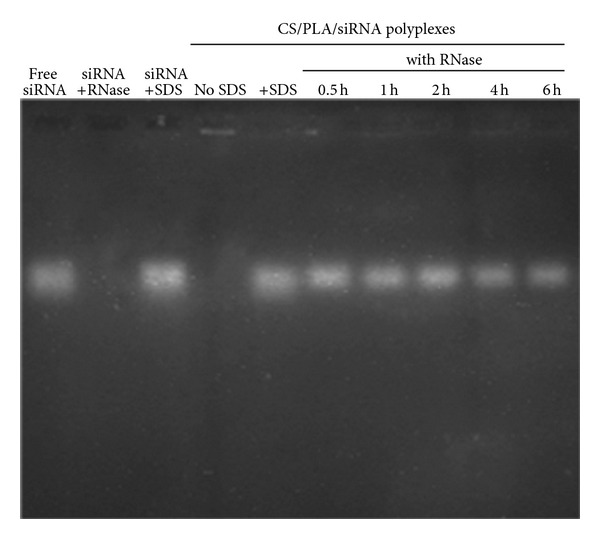
RNase stability of siRNA in the CS/PLA/siRNA complexes. Naked siRNA was a negative control. The complexes were incubated with RNase A (5 mIU/**µ**g siRNA) at 37°C for 6 h. The reaction was inactivated at 70°C and was released by SDS. The remaining siRNA was analyzed by agarose gel electrophoresis.

RNA degradation by nuclease attack is one of the most important barriers for siRNA delivery and eventually reduces the gene silencing efficacy [30]. Therefore, the integrity of siRNA in the complexes in the presence of RNase must be maintained, for example, by protection ability of vehicle. From the results (Figure 6), naked siRNA was entirely digested upon the incubation with RNase A at 37°C for 10 min. On the other hand, siRNA which was incorporated in the polyplexes of CS combined with PLA at CS/PLA/siRNA weight ratio of 5 : 0.5 : 1 remained intact for a period of at least 6 h. This finding clearly indicated the ability of CS/PLA to protect siRNA from enzymatic degradation and implied the enhanced siRNA if it is transferred *in vivo*.

### 3.4. Cytotoxicity Results

**Figure 7:**
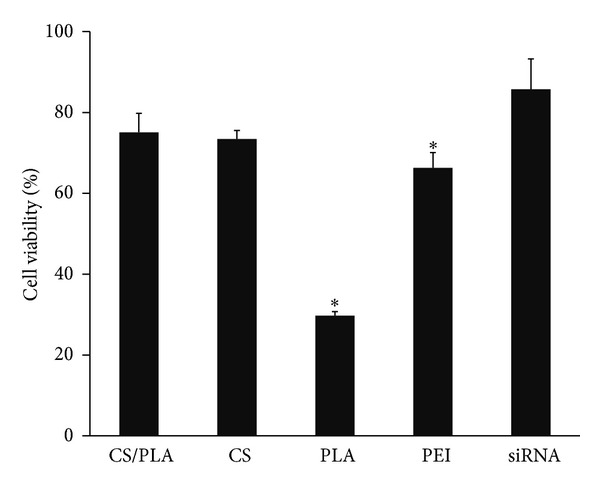
Comparison of cell viability of polymers/siRNA complexes in HeLa cells stably expressing EGFP. The weight ratios of CS/PLA/siRNA, CS/siRNA, PLA/siRNA, and PEI (25 kDa)/siRNA were 5 : 0.5 : 1, 5 : 1, 5 : 1, and 2 : 1, respectively. Data are plotted in mean ± SD (*n* = 3). *Significantly different from the group treated with CS/PLA/siRNA complexes.

As shown in Figure 7, the average viability of cells treated with CS/PLA/siRNA (5 : 0.5 : 1) polyplexes was about 75% and it was not statistically different from that treated with naked siRNA or CS/siRNA (5 : 1) polyplexes. However, the siRNA polyplexes formulated by using CS combined with PLA showed lower cytotoxicity than PEI/siRNA (2 : 1). Furthermore, the result clearly demonstrated that the preparation of polyplexes as CS/PLA/siRNA which could lower the quantity of PLA used in the formulation from 5 to 0.5 helped decrease the cytotoxicity of CS/PLA/siRNA (5 : 0.5 : 1) compared to that of PLA/siRNA (5 : 1).

## 4. Conclusion

 CS combined with PLA at the optimal ratio was able to form positively charged, nanosized polyplex with siRNA and successfully delivered siRNA to the cells *in vitro*. Different from CS/siRNA and PLA/siRNA polyplexes which had very low transfection efficiency at physiological pH (7.4), CS/PLA/siRNA polyplex formed by using sufficient amount of CS (i.e., 5 : 1 CS/siRNA) together with noncytotoxic level of PLA (i.e., 0.1 : 1 PLA/siRNA) could silence the gene satisfactorily at both pH 7.4 and 6.4, unaffected by the presence of serum. Also, CS combined with PLA protected the delivered siRNA from RNase degradation. Since CS combined with PLA is proven safe and easy to prepare without complicated or time-consuming chemical conjugation steps, it is a promising vehicle efficiently used for siRNA delivery.
